# Isolated aneurysmal disease as an underestimated finding in individuals with *JAG1* pathogenic variants

**DOI:** 10.1002/humu.24433

**Published:** 2022-07-16

**Authors:** Jotte Rodrigues Bento, Alice Krebsová, Ilse Van Gucht, Irene Valdivia Callejon, An Van Berendoncks, Pavel Votypka, Ilse Luyckx, Petra Peldova, Steven Laga, Marek Havelka, Lut Van Laer, Pavel Trunecka, Nele Boeckx, Aline Verstraeten, Milan Macek, Josephina A. N. Meester, Bart Loeys

**Affiliations:** ^1^ Centre of Medical Genetics Antwerp University Hospital/University of Antwerp Antwerp Belgium; ^2^ Department of Cardiology, Center for Inherited Cardiovascular Disorders Institute for Clinical and Experimental Medicine (IKEM) Prague Czech Republic; ^3^ Department of Cardiology Antwerp University Hospital/University of Antwerp Antwerp Belgium; ^4^ Department of Biology and Medical Genetics, Second Faculty of Medicine Charles University and Motol University Hospital Prague Czech Republic; ^5^ Department of Human Genetics Radboud University Medical Center Nijmegen The Netherlands; ^6^ Department of Cardiac Surgery Antwerp University Hospital/University of Antwerp Antwerp Belgium; ^7^ Department of Hepatology and Gastroenterology Transplant Center of Institute for Clinical and Experimental Medicine (IKEM) Prague Czech Republic

**Keywords:** Alagille syndrome, intracranial aneurysm, *JAG1*, thoracic aortic aneurysm

## Abstract

Pathogenic variants in *JAG1* are known to cause Alagille syndrome (ALGS), a disorder that primarily affects the liver, lung, kidney, and skeleton. Whereas cardiac symptoms are also frequently observed in ALGS, thoracic aortic aneurysms have only been reported sporadically in postmortem autopsies. We here report two families with segregating *JAG1* variants that present with isolated aneurysmal disease, as well as the first histological evaluation of aortic aneurysm tissue of a *JAG1* variant carrier. Our observations shed more light on the pathomechanisms behind aneurysm formation in *JAG1* variant harboring individuals and underline the importance of cardiovascular imaging in the clinical follow‐up of such individuals.

1

*JAG1* serves as one of five Notch interacting surface ligands and is ubiquitously expressed in the embryo, while expression in adult tissue is restricted to the heart, placenta, pancreas, prostate, and large arteries (Carithers & Moore, 2015; Grochowski et al., 2016). Given that the Notch pathway is a highly conserved signaling cascade engaged in normal development of fetal organs and in cell fate decisions in postnatal life, it is not unexpected that variants in Notch pathway components can cause various diseases (Grochowski et al., 2016). Alagille syndrome (ALGS) is one such rare heritable disorder that is predominantly caused by loss‐of‐function variants in *JAG1* (in 94.3% of ALGS patients [of which 87% are haplo‐insufficient], another 2.5% has *NOTCH2* variants, 3.2% of patients remains unknown) and has an estimated incidence of 1:30,000 live births (Gilbert et al., 2019). ALGS is clinically diagnosed when three out of seven typical clinical features are observed, but due to marked variability (even within families), patients can remain undiagnosed. A molecular diagnosis is therefore the key to adequate patient management (Saleh et al., 2016). Hepatic abnormalities, with intrahepatic duct deficiency leading to cholestasis as the most prevalent manifestation, are observed in up to 100% of patients; posterior embryotoxon has a prevalence of approximately 80%–90%; and cardiac structural changes such as Tetralogy of Fallot and peripheral pulmonary artery stenosis are present in over 90% of patients (Saleh et al., 2016; Spinner et al., 1993). Renal disease, distinct facial features, and butterfly and hemi‐vertebrae are also reported frequently. Furthermore, noncardiac vascular complications were noted to be a significant part of the clinical spectrum (estimated in 34% of ALGS patients), with intracranial aneurysm and hemorrhage as the most frequent finding (Kamath et al., 2004; Saleh et al., 2016; Spinner et al., 1993).

Using whole‐exome sequencing on the DNA of a fetus presenting with left hypoplastic heart syndrome and left renal agenesis (Figure 1a, IV:4), a missense variant in *JAG1* (NM_000214:c.2242T>C, p.Cys748Arg, ClinVar accession VCV001172525.1) was discovered. The variant (i) replaces a highly conserved and critical cysteine residue that is part of a disulfide bridge in one of the 14 EGF repeats, which are important for Notch receptor binding; (ii) is absent from control databases (including GnomAD); (iii) has not been previously reported; and (iv) was the only relevant pathogenic variant identified in this patient (Ashkenazy et al., 2016; Grochowski et al., 2016; Karczewski et al., 2020). The *JAG1* variant found in the fetus was inherited from the mother (Figure 1a, III:6, proband, 35 years old), who underwent surgery at the age of 10 for aortic coarctation, a feature that has been linked to ALGS before (Kamath et al., 2004). Examination of the mother and her siblings revealed absence of typical ALGS manifestations. However, in both the monozygotic twin sister (Figure 1a, III:8) and brother (36 years old, Figure 1a, III:11), thoracic aortic aneurysms (TAA) were discovered with a diameter of 40 mm (*Z*‐score of 3.6) and 58 mm (*Z*‐score of 8.6), respectively, urging the brother to undergo immediate Bentall surgery with mechanical aortic valve replacement. Histological examination of aorta obtained during surgery showed fragmentation of elastic fibers with marked decrease in elastin content (Figure 2a,d). Collagen was drastically increased and disorganized compared to a healthy control sample (Figure 2c,b,e). Since the histological phenotype in our patient resembles that in Marfan syndrome and Loeys‐Dietz syndrome patients (Maleszewski et al., 2009) and dysregulated transforming growth factor‐β (TGF‐β) signaling is a key feature of these syndromes, we also studied whether phosphorylated SMAD2 (pSMAD2), a downstream marker of TGF‐β activity, is increased here too. Indeed, we qualitatively observed that more nuclei were positive for pSMAD2 (Figure 2f) compared to a control sample (Figure 2c). The father of the proband (Figure 1a, II:7, aged 69) harbored the variant and did not show any features besides aortic wall calcifications. Segregation analysis revealed presence of the variant in three siblings (II:2, II:5, and II:6; Figure 1a). Additional aneurysm gene panel testing in individual III:11 did not reveal additional (likely) pathogenic variants. Of note, the uncle of the proband (II:2 63 years old) suffered from multiple intracranial aneurysms (IA) and subarachnoid bleeding from ruptured arteria communicans anterior aneurysm. The aunt (II:5, aged 65) presented with bicuspid aortic valve but normal aortic measurements (sinus 34 mm, aorta ascendens 34 mm). The second aunt (II:6; aged 60) had normal echocardiographic evaluation. This marked interfamilial phenotypic variability reflects the significant and repeatedly described range in disease expression of ALGS (Gilbert et al., 2019; Guegan et al., 2012). Individual IV:6 had a normal echocardiographic evaluation, but she is only 3 years old. Intriguingly, apart from the cardiac and renal phenotype of the fetus (Figure 1a, IV:4), none of the patients showed any of the typical ALGS features.

**Figure 1:**
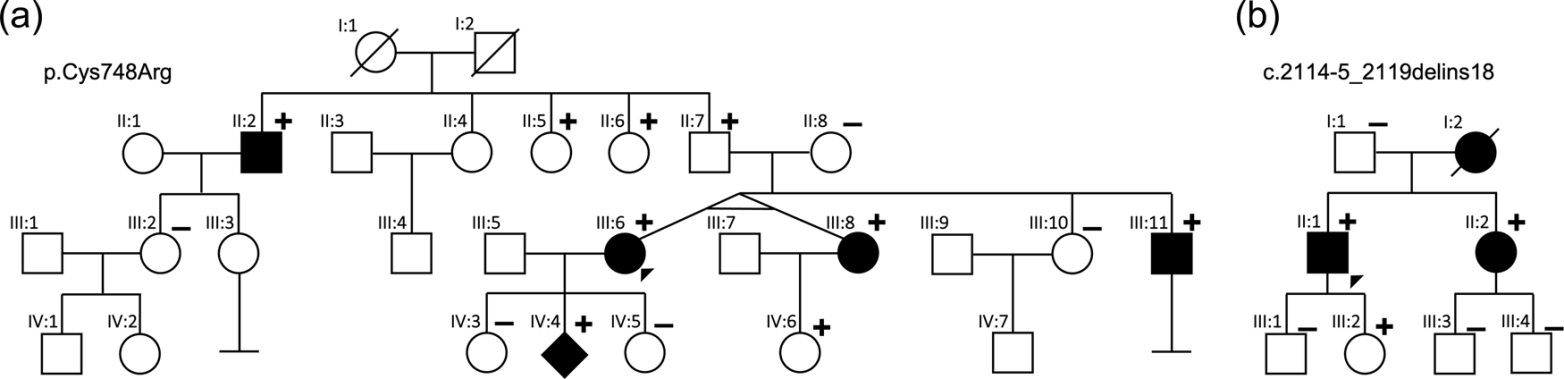
Pedigrees of two families with *JAG1* variants. (a) family with p.Cys748Arg variant; (b) family with the c.2114‐5_2119delins18 variant.

**Figure 2:**
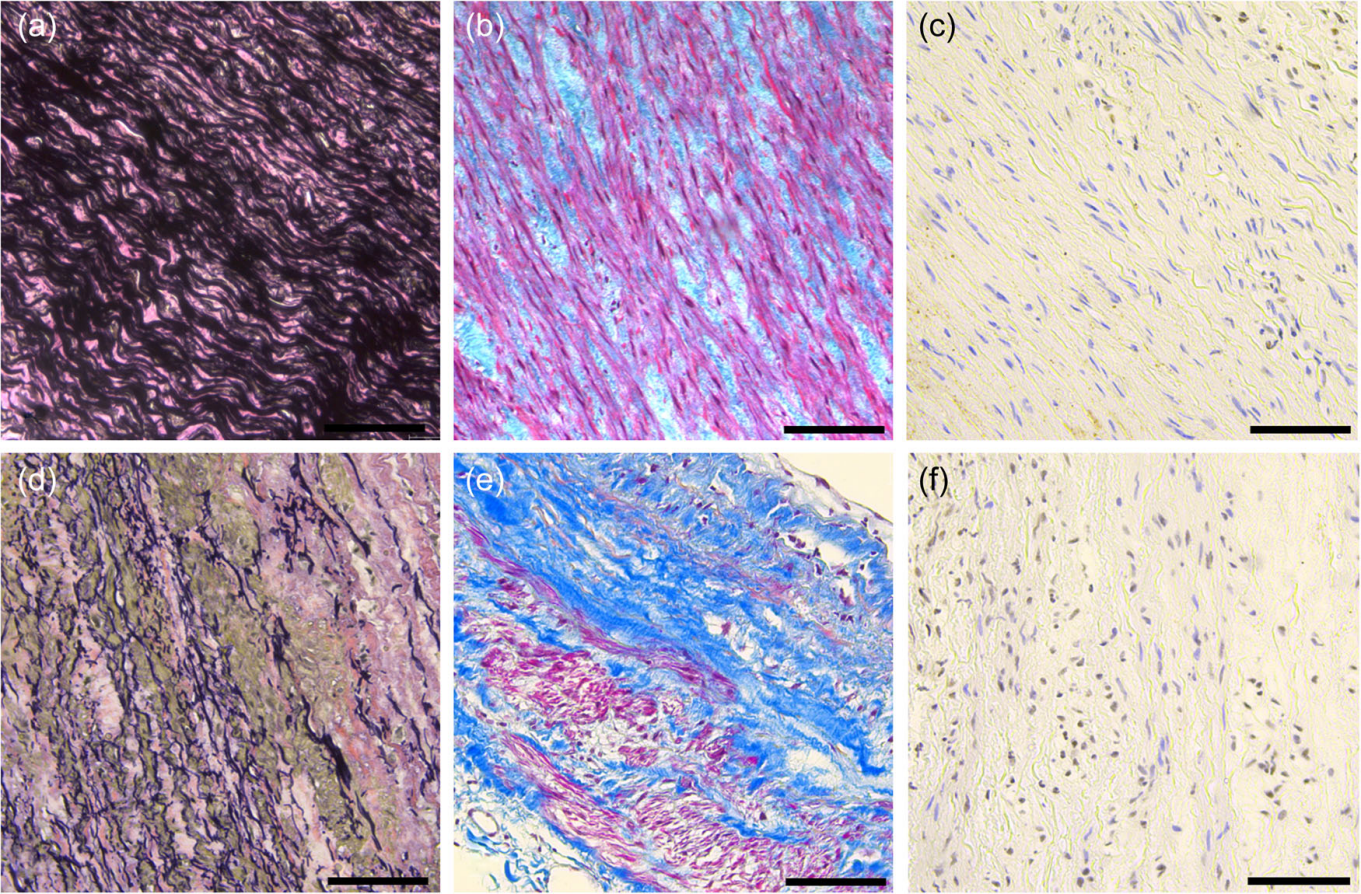
Histological staining of elastin (a, d), collagen (b, e), and phosphorylated SMAD2 protein (pSMAD2, brown nuclear staining, c, f) of aortic tissue of a healthy control (top) and patient A:III:11 (bottom). Scale bar: 100 µm.

In a second family, sequencing of a custom‐made cardiac/aortic conditions‐related gene panel revealed deletion‐insertion variant of *JAG1* in the 42‐year‐old male TAA proband (Figure 1b, II:1). The variant affects the splice acceptor site and first nucleotides of exon 17 (NM_000214.2: c.2114‐5_2119delins18, Clinvar accession SCV002515924). The variant was not reported before and RNA analysis of the proband revealed in‐frame skipping of exon 17 (Supporting Information: Figure S1). No other relevant genetic variants were identified. At age 24 years, the patient underwent Bentall surgery with mechanical aortic valve replacement because of an incidental finding of aortic aneurysm (at age 22, ascending aorta 60 mm, *Z*‐score of 10). Other family members, including the proband's sister who also harbored the deletion‐insertion variant, also presented with severe vascular disease. The mother of the proband (Figure 1b, I:2) underwent preventive surgery with aortic replacement for TAA at the age of 61 and died at the age of 63 of an intracranial bleeding associated with IA. The proband's sister (Figure 1b, II:2) suffered multiple IAs (left basilary and cerebral artery, arteria cerebri posterior, bilateral arteria cerebri media) and subarachnoid bleeding events, but showed normal aortic dimensions. The proband's daughter (Figure 1b, III:2—age 13 years), which harbors the pathogenic variant, had a normal total body MR angiography. In accordance with the first family, patients in family B (Figure 1b, II:2, II:1, II:2) did not present with typical ALGS hepatic, ocular or skeletal characteristics.

We are the first to report two ALGS families with isolated aneurysmal disease lacking other ALGS pathognomonic clinical characteristics (such as the hepatic abnormalities, posterior embryotoxon, and cardiac structural changes described above). In the current ALGS literature there is no notion of TAA in the vast majority of ALGS patient descriptions, but systematic aortic evaluation might not be performed on a routine basis. TAA has been reported before in only four ALGS patients, three of which were discovered at autopsy (Kamath et al., 2004; Molinero‐Herguedas et al., 2008). In all four, at least one typical clinical ALGS manifestation was present: in the patient reported by Molinero‐Herguedas et al., TAA was discovered during reexamination of an (undefined) cardiopathy and three patients reported in Kamath et al. had characteristic facial features, but due to discovery at autopsy no details on other organ system involvement were available. Similar for IA in ALGS, which has an estimated prevalence of 14%, patients previously reported always presented accompanying cholestatic liver disease and cardiac defects (Kamath et al., 2004).

For the first time, histological evaluation of aortic tissue of a TAA patient with a *JAG1* variant was performed, revealing an unexpected phenotype with elastin degradation and abnormal collagen deposition. Fibrosis has been associated with *JAG1* gain‐of‐function rather than loss‐of‐function in fibrotic kidney disease, where excessive Notch signaling is known to induce disproportionate expression of extracellular matrix (ECM) components (Hu et al., 2015). Additionally, in a mouse model of abdominal aortic aneurysm, pharmacological inhibition of Notch signaling resulted in regression of aneurysm along with a reduction in elastic fiber fragmentation and collagen deposition (Sharma et al., 2019). On the other hand, it has been observed that dysfunction of Notch signaling mitigates epithelial‐to‐mesenchymal transition (EMT), an indispensable process during cardiovascular development and repair mechanisms. Additionally, contractile markers of vascular smooth muscle cells (VSMC) are downregulated upon Notch impairment, suggesting that the VSMC in the aortic media of ALGS patients adopt a synthetic phenotype, characterized by increased ECM synthesis and elastolysis due to secretion of matrix metalloproteases. Resulting tissue damage and subsequent failure to sufficiently dampen hemodynamical pressure could trigger TGF‐β signaling, which is expressed by increased pSMAD2 in our patient. Knowing that TGF‐β is a well‐known mediator of fibrosis and a driver of metalloprotease activity, a destructive cycle is initiated, leading to aortic wall degradation (Jones et al., 2009; Kostina et al., 2016; Zavadil, Cermak, Soto‐Nieves, & Bottinger, 2004). Further functional studies will be necessary to determine the true sequence of events of ALGS‐related TAAD.

With the current report of these patients with significant *Z*‐scores (3.6–8.6) and intracranial hemorrhaging at a relatively young age without obvious ALGS findings, we want to urge clinicians to include *JAG1* (and *NOTCH2*) in genetic screening panels for TAA and IA as this is not yet the case in most clinics—most probably due to insufficient clinical evidence (Renard et al., 2018), which we now provide. Furthermore, we anticipate TAA might be an underestimated finding in ALGS and we, therefore, advise to early and thoroughly examine the vascular system in such patients, with focus on intracranial and thoracic aorta imaging, since vascular events are estimated to account for over 30% of ALGS mortality (Ayoub & Kamath, 2020; Kamath et al., 2004). A systematic large‐scale ALGS cohort investigation could straighten out the true significance of aortopathy in ALGS.

## CONFLICT OF INTEREST

The authors declare no conflict of interest.

## Supporting information


**Figure S1**: Splicing analysis of the insertion‐deletion variant encompassing the splice acceptor site and first nucleotides of exon 17 (NM_000214.2: c.2114‐5_2119delins18).

Supplementary information.
