# Supplementary material for: Isolated aneurysmal disease as an underestimated finding in individuals with JAG1 pathogenic variants
Source: Hum Mutat. 2022 Jul 16;43(12):1824–8. doi: 10.1002/humu.24433 (PMC10084246; doi:10.1002/humu.24433)
Supplement: Supplementary file 2 — Supplementary information. [file HUMU-43-1824-s002.docx]

**Materials and methods**

**Whole exome sequencing:**

DNA of the fetus and parents (Figure 1A IV:4) was subjected to whole exome sequencing (WES) according to the manufacturer's protocols. DNA was fragmented using the Covaris M220 Focused-ultrasonicator and subsequently prepped with the TruSeq DNA Sample Preparation kit (Illumina). The SeqCap EZ Human Exome Library v3.0 kit was used for enrichment (NimbleGen, Roche) and finally, sequencing was carried out on a HiSeq 2000 (Illumina). Analysis of WES data was performed using VariantDB (Vandeweyer et al., 2014). Sanger sequencing was performed to confirm the presence of the variant in the fetus and subsequently to perform segregation analysis in the other family members. A PCR reaction using GOTaq polymerase (Promega) was followed by sequencing using the ABI BigDye Terminator V3.1 Cycle Sequencing kit (Applied Biosystems) and the ABI 3130 Genetic Analyzer (Applied Biosystems).

DNA of members of family B (II:1, II:2 and III:1-4) was subjected to a custom-made panel comprising 100 cardiac/aortic conditions-related candidate genes (SOPHiA Genetics, Switzerland) according to the manufacturer's protocols. The sequencing was carried out on a MiSeq (Illumina). Variant prioritization was performed by Sophia DDM software (Sophia Genetics, Switzerland). The evaluation was carried out by bioinformatics tools integrated in Varsome Clinical software (Saphetor SA, Switzerland). Presence of the detected variant was validated by Sanger DNA sequencing and family segregation was performed.

**Splicing analysis of family B**

RNA analysis was performed from blood of the proband drawn into a PAXgene Blood RNA Tube, followed by RNA isolation and a reverse transcriptase reaction followed by Sanger sequencing covering *JAG1* exons 16-18 (primers: F: GGCCTACTGTGAAACCAATATT, R:TCATTGGTATTCTGAGCACAG).

**Elastic fiber, collagen and pSMAD2 staining**

Collected aortic tissue was embedded in paraffin and cut into 5µm thick sections. Elastic fiber integrity and collagen content were assessed using Verhoeff Van Gieson (Sigma-Aldrich) and Trichrome Masson’s (Sigma-Aldrich), respectively, according to manufacturer’s instructions. pSMAD2 was visualized using immunohistochemistry. Deparaffinization in toluene (5 minutes) was followed by rehydration in 100%, 90%, 70% and 50% ethanol solutions, after which sections were subjected to 3% hydrogen peroxide followed by incubation in trypsin for 10 minutes at 37 °C (Sigma-Aldrich, 93615-25G). Sections were heated in citrate buffer for 10 minutes at 90°C and blocked for 20 minutes at room temperature (RT) with goat serum (Vector-lab consult). A primary pSMAD2 antibody (3101, Cell Signaling; 1:5000) was incubated overnight using a concentration of 1:5000 and the following day, the sections were incubated with the secondary antibody (30014 secondary goat anti-rabbit IgG, Vector; 1:200) for 30 min and subsequently with an avidin-biotinylated complex for 1 hour, both at RT (Vectastain ABC kit, Vector Laboratories). 3,3-Diaminobenzidine tetrahydrochloride hydrate (DAB) chromogen (Sigma-Aldrich) was used as the substrate and Hematoxylin as counterstaining. All pictures were acquired using a Leica DMi8 inverted microscope, at a 20x magnification.

Vandeweyer, G., Van Laer, L., Loeys, B., Van den Bulcke, T., & Kooy, R. F. (2014). VariantDB: a flexible annotation and filtering portal for next generation sequencing data. *Genome Med, 6*(10), 74. <https://doi.org/10.1186/s13073-014-0074-6>
