# Supplementary material for: Isolated aneurysmal disease as an underestimated finding in individuals with JAG1 pathogenic variants
Source: Hum Mutat. 2022 Jul 16;43(12):1824–8. doi: 10.1002/humu.24433 (PMC10084246; doi:10.1002/humu.24433)
Supplement: Supplementary file 1 — Figure S1: Splicing analysis of the insertion‐deletion variant encompassing the splice acceptor site and first nucleotides of exon 17 (NM_000214.2: c.2114‐5_2119delins18). [file HUMU-43-1824-s001.pdf]

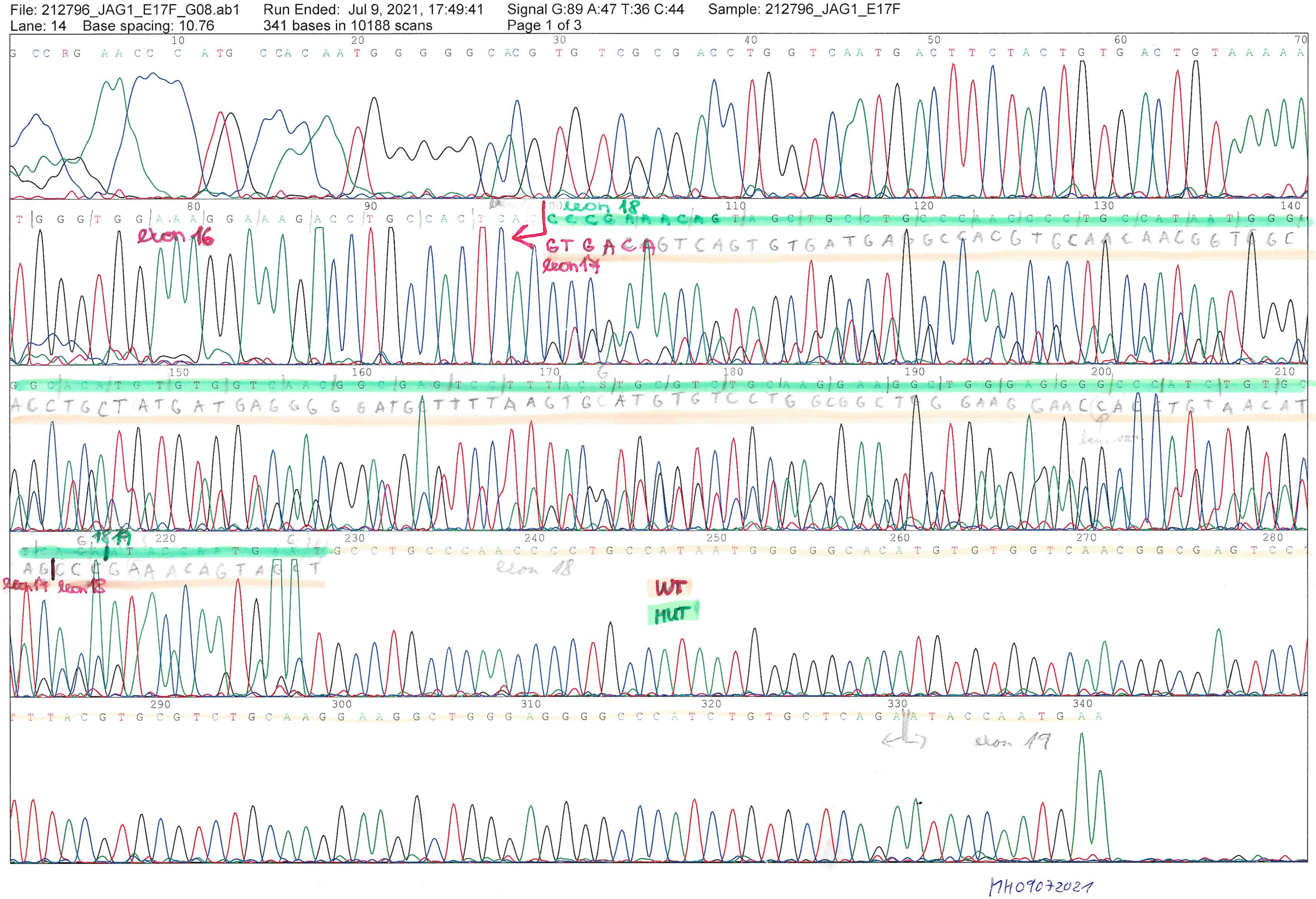

Figure S1: Splicing analysis of the insertion-deletion variant encompassing the splice acceptor site and first nucleotides of exon 17 (NM_000214.2: c.2114-5_2119delins18).
